# Capsid Integrity Detection of Enteric Viruses in Reclaimed Waters

**DOI:** 10.3390/v16060816

**Published:** 2024-05-21

**Authors:** Pablo Puchades-Colera, Azahara Díaz-Reolid, Inés Girón-Guzmán, Enric Cuevas-Ferrando, Alba Pérez-Cataluña, Gloria Sánchez

**Affiliations:** VISAFELab, Department of Preservation and Food Safety Technologies, Institute of Agrochemistry and Food Technology, IATA-CSIC, Av. Agustín Escardino 7, 46980 Valencia, Spain; pablopc@iata.csic.es (P.P.-C.); azahara.diaz@iata.csic.es (A.D.-R.); alba.perez@iata.csic.es (A.P.-C.)

**Keywords:** enteric viruses, virus contamination indicator, capsid integrity (RT)-qPCR, wastewater, reclaimed water

## Abstract

Climate change, unpredictable weather patterns, and droughts are depleting water resources in some parts of the globe, where recycling and reusing wastewater is a strategy for different purposes. To counteract this, the EU regulation for water reuse sets minimum requirements for the use of reclaimed water for agricultural irrigation, including a reduction in human enteric viruses. In the present study, the occurrence of several human enteric viruses, including the human norovirus genogroup I (HuNoV GI), HuNoV GII, and rotavirus (RV), along with viral fecal contamination indicator crAssphage was monitored by using (RT)-qPCR methods on influent wastewater and reclaimed water samples. Moreover, the level of somatic coliphages was also determined as a culturable viral indicator. To assess the potential viral infectivity, an optimization of a capsid integrity PMAxx-RT-qPCR method was performed on sewage samples. Somatic coliphages were present in 60% of the reclaimed water samples, indicating inefficient virus inactivation. Following PMAxx-RT-qPCR optimization, 66% of the samples tested positive for at least one of the analyzed enteric viruses, with concentrations ranging from 2.79 to 7.30 Log_10_ genome copies (gc)/L. Overall, most of the analyzed reclaimed water samples did not comply with current EU legislation and contained potential infectious viral particles.

## 1. Introduction

In recent years, wastewater-based epidemiology has become a useful tool for tracking pathogens with notable epidemiological implications. Recent studies have successfully applied this approach to detect a range of viruses, including the re-emergence of poliovirus in New York [1], the tracking of severe acute respiratory syndrome coronavirus 2 (SARS-CoV-2) [2,3], monkeypox virus [4], and human enteric viruses [5,6]. Furthermore, the analysis of viruses in water serves to evaluate the safety of aquatic environments and determine the suitability of reclaimed water for various purposes, such as recreational, agricultural, and industrial applications [7].

Human enteric viruses, primarily transmitted through the fecal–oral route (direct contact with infected individuals, or ingestion of contaminated food and water), are responsible for viral gastroenteritis, hepatitis, and other diseases [8]. Furthermore, both symptomatic and asymptomatic individuals can shed up to 10^13^ viral particles per gram of stool [9].

Wastewater treatment systems are designed to reduce the concentration of organic matter, suspended solids, and pathogenic microorganisms [10]. However, enteric viruses tend to be more persistent in the environment and resistant to the removal and disinfection processes typically applied by wastewater treatment plants (WWTPs) [7,11]. As a result, achieving water quality with the complete removal of viral particles from reclaimed water and preventing their presence in environmental samples has proven challenging [10,12,13,14,15,16,17,18].

Considering the current water scarcity and adverse climatic conditions, it is imperative to reuse available water resources, particularly reclaimed water for agricultural purposes, as agriculture consumes a high proportion of water [19]. However, inappropriate use of reclaimed water has led to outbreaks of viral infectious diseases worldwide [5], and the reuse of wastewater in agriculture can pose health risks associated with the consumption of fresh vegetables and berries [20]. *Escherichia coli* and other fecal indicator bacteria are commonly used for assessing the microbial quality of WWTP effluent; however, many studies have demonstrated that these methods may not accurately represent the spectrum of pathogens present in feces, particularly human enteric viruses [16]. Therefore, the European Regulation (EU) 2020/741 [21] has established a minimum requirement of ≥6 Log_10_ reduction in the concentration of F-specific coliphages, somatic coliphages, or total coliphages for the use reclaimed water for agricultural irrigation. In addition to fecal indicator bacteria and coliphages, the use of crAssphage has been proposed in recent years to estimate viral contamination in environmental waters and to assess the efficiency of viral removal during wastewater treatment [6,7,22,23,24].

Currently, real-time polymerase chain reaction (qPCR) is the method of choice to monitor human viral pathogens in wastewater and environmental samples [6,12]. However, qPCR methods cannot discriminate between infectious viruses, inactivated viruses, or free viral genomes. To address this limitation, samples can be pretreated with intercalating dyes such as propidium monoazide (PMA), ethidium monoazide, or platinum compounds. These dyes selectively allow the detection of viruses with intact capsids, providing a more accurate assessment of viral infectivity [25,26,27,28].

Thus, in this study influent wastewater and reclaimed water samples were analyzed for the presence of human pathogenic viruses over ten months using rapid molecular methods. Additionally, an optimized PMAxx-RT-qPCR method was developed to infer viral infectivity in both sample types, particularly in reclaimed water intended for irrigation. This study also aimed to investigate the correlation between crAssphage and somatic coliphages with the presence of human enteric viruses.

## 2. Materials and Methods

### 2.1. Sampling Site and Sample Collection

Influent wastewater (n = 30) and reclaimed water (n = 30) samples were collected from May 2022 to March 2023 from a WWTP in the Comunitat Valenciana (Spain) serving 170,000 inhabitants. In the sampled wastewater treatment plant, reclamation processes include tertiary UV treatment combined with chlorination. Grab samples (200 mL) were collected in sterile HDPE plastic containers (Labbox Labware, Barcelona, Spain), placed on ice, and transported to the laboratory. Upon arrival, they were kept refrigerated at 4 °C and concentrated within 24 h.

### 2.2. Somatic Coliphages Determination

To quantify the levels of somatic coliphages, an aliquot of the water samples was filtered through sterile filters with a pore size of 0.45 μm. The commercial Bluephage Easy Kit for Enumeration of Somatic Coliphages (Bluephage S.L., Barcelona, Spain) was used according to the manufacturer’s instructions.

### 2.3. Virus Concentration

Influent wastewater and reclaimed water samples were artificially inoculated with approximately 7 Log_10_ genome copies (gc)/L of mengovirus (MgV) strain vMC0 (CECT 100000) as a process control. Samples were concentrated using an aluminum hydroxide adsorption–precipitation method^7^. In brief, 200 mL of each sample was adjusted to pH 6.0, and an Al(OH)_3_ precipitate was formed by adding 1 part of 0.9 N AlCl_3_ solution to 100 parts of the sample. After adjusting the pH back to 6.0, the sample was mixed using an orbital shaker for 15 min at room temperature (RT). The viruses were then collected by centrifugation, and the pellet was resuspended in 10 mL of 3% beef extract pH 7.4. After shaking for 10 min, the water concentrate was recovered by centrifugation, resuspended in 1 mL of PBS, and stored at −80 °C.

### 2.4. Nucleic Acid Extraction, Detection and Quantification

Nucleic acid extraction from influent wastewater and reclaimed water concentrates was performed using the Maxwell^®^ RSC Instrument (Promega, Madison, WI, USA) with the Maxwell RSC Pure Food GMO and authentication kit (Promega) and the “Maxwell RSC Viral Total Nucleic Acid” running program [29,30].

For viral detection and quantification, different kits and instruments were used depending on the targeted virus. The One Step PrimeScript™ RT-PCR Kit (Perfect Real Time, Takara Bio Inc., San Jose, CA, USA) was used for the detection and quantification of the MgV. The RNA UltraSense One-Step kit (Invitrogen, Waltham, MA, USA) was used for the detection of human norovirus (HuNoV) genogroup I (GI), HuNoV GII, and rotavirus (RV) as previously described [7]. The QuantStudio™ 5 Real-Time PCR (Applied Biosystem, Waltham, MA, USA) and the LightCycler^®^ 480 instrument (Roche Diagnostics, Basel, Switzerland) were used for the PCR amplification. The qPCR Premix Ex Taq™ kit (Takara Bio Inc.) was used for the detection of crAssphage [31]. Primers, probes, and (RT)-qPCR conditions used in the study are listed in Supplementary Table S1. Moreover, undiluted and 10-fold diluted nucleic acid extracts were tested in duplicate to check for inhibitors. Different controls were used in all assays: negative extraction control consisting of PBS; whole process control to monitor the process efficiency of each sample (MgV); and positive (reference material) and negative (RNase-free water) (RT)-qPCR controls. Standard DNA material for crAssphage, HuNoV GI, HuNoV GII, and RV for standard curve generation relied on customized gBlock gene fragments (Integrated DNA Technologies, Coralville, IA, USA).

### 2.5. Viral Capsid Integrity Assay in Sewage Samples and Optimization in Influent Wastewater

To assess the integrity of viral capsids on sewage samples, a protocol based on capsid integrity to PMAxx was evaluated [32]. Briefly, samples were placed in DNA LoBind 1.5 mL tubes (Eppendorf, Hamburg, Germany), and the photoactivable dye PMAxx^TM^ (Biotium, Fremont, CA, USA) was added to 300 µL of each concentrated influent wastewater sample at 100 µM final concentration along with 0.5% Triton 100-X (Thermo Fisher Scientific, Valencia, Spain) and then incubated in the dark at RT for 10 min at 150 rpm. Later, samples were photoactivated for 15 min using a Led-Active Blue system (GenIUL, Barcelona, Spain), and nucleic acid extraction was carried as described above. Due to the initially observed underperformance of this procedure, the capsid integrity assay was further optimized by diluting the concentrates in PBS (5-fold and 2-fold) and incorporating an additional sample incubation and photoactivation cycle. PMAxx-RT-qPCR optimization assays were conducted targeting HuNoV GI, HuNoV GII, and RV in influent wastewater samples exposed or not to thermal inactivation at 99 °C for 5 min.

### 2.6. Statistical Analysis

Statistical analyses were performed using GraphPad Prism version 5.0 (GraphPad, La Jolla, CA, USA). Data were checked for normality distribution using the Shapiro–Wilk normality test. Non-parametric tests, such as the Kruskal–Wallis test with Dunn’s multiple comparisons post-test and Spearman ρ coefficient non-parametric correlation test, were used to compare viral loads between influent wastewater and reclaimed water, assess distribution of enteric viruses, and determine the correlation between viral titers. A *t*-test was used to analyze differences in viral removal after capsid integrity treatment. The significance level was set at a *p*-value cut-off of 0.05.

## 3. Results and Discussion

### 3.1. Prevalence of Enteric Viruses, crAssphage, and Somatic Coliphages in Influent Wastewater and Reclaimed Water Samples

**Figure 1:**
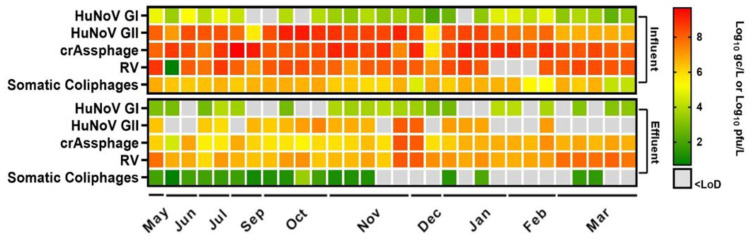
Presence of human enteric viruses, crAssphage, and somatic coliphages in influent and effluent wastewater samples over 10 months. Each colored square represents mean Log10 genome copies (gc)/L or Log10 plaque-forming units (pfu)/L values. Abbreviations: human norovirus genotype I (HuNoV GI), human norovirus genotype II (HuNoV GII), rotavirus (RV), limit of detection (LoD).

The relevance of water as a vector of viral diseases has been known for decades; however, due to climate change and water scarcity, reclamation of wastewater is of the utmost importance. Thus, in this study, influent wastewater and reclaimed water were analyzed over 10 months to determine the presence of HuNoV GI, HuNoV GII, and RV together with recent proposed viral fecal contamination indicator, crAssphage and total somatic coliphages (Figure 1).

The recovery of the process control, MgV, ranged from 8.08% to 63.64% for influent wastewater samples and from 11.72% to 99.20% for reclaimed water samples (Supplementary Table S2). Thus, the obtained results were validated based on the criteria outlined in ISO 15216-1:2017 [33], where a recovery control of ≥1% is required. Considering the characteristics of the samples and the study’s objectives, viral titers were not adjusted based on the recovery of the process control, as back-calculation is not recommended [34].

The average viral concentrations in influent wastewater (n = 30) were 4.11 ± 0.62 (26/30), 7.87 ± 0.97 (30/30), and 8.11 ± 1.31 (27/30) Log_10_ gc/L for HuNoV GI, HuNoV GII and RV, respectively (Figure 1). Haramoto and collaborators [34] summarized the average concentrations of HuNoV GI, HuNoV GII, and RV in different environmental water samples, and our results are consistent with those findings, except for RV, for which we recorded higher levels. Additionally, these findings align with those reported by Stobnicka and collaborators [17], where HuNoV GII was the most prevalent enteric virus, followed by HuNoV GI and RV. Similar results have also been reported by other authors [7,35], showing a higher concentration of RV followed by HuNoV GII and HuNoV GI in influent wastewater. However, Randazzo et al. [26] described lower levels for RV (5.41–6.52 Log PCR units (PCRU)/L).

**Figure 2:**
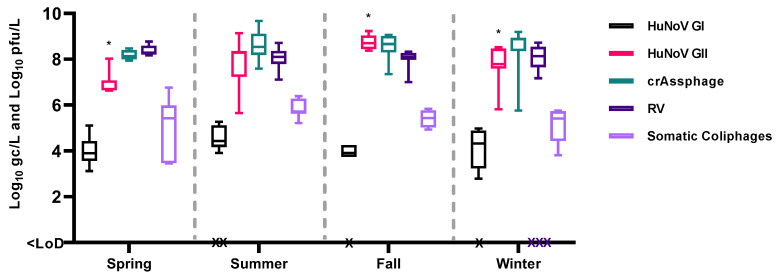
Temporary distribution of enteric viruses, crAssphage and somatic coliphages on influent samples. Bars represent mean Log_10_ genome copies (gc)/L or Log_10_ plaque-forming units (pfu)/L values. * *p* < 0.05 between seasons for HuNoV GII. Abbreviations: human norovirus genotype I (HuNoV GI), human norovirus genotype II (HuNoV GII), rotavirus (RV), limit of detection (LoD).

There are few studies that have analyzed the distribution of enteric viruses in environmental samples over long periods of time [12,36,37], and particularly in sewage [7,13,34]. The viral concentrations obtained over ten months and distributed across the study by season are represented in Figure 2.

In influent wastewater, statistically higher levels of HuNoV GII were observed during the fall season (*p* < 0.05). These trends align with previously findings [14,15] that also reported higher levels of HuNoV GI and GII in the cold months (October–March), with HuNoV GII being more prevalent than HuNoV GI [38,39,40]. However, considering the duration of this study, the term seasonality may not be fully applicable. To accurately assess the impact of climate on the distribution of enteric viruses in environmental samples, more extensive and longer-term studies, spanning at least three years, are deemed necessary.

Regarding viral fecal indicators, crAssphage showed the highest concentrations, which ranged from 5.71 to 9.67 Log_10_ gc/L (30/30) in influent wastewater. Wu et al. [36] reported values ranging from 7.20 to 8.96 Log_10_ gc/L on influent wastewater, which aligns with concentrations reported in other studies from Italy, US, and Japan [41,42,43]. The concentration of crAssphage in influent wastewater can reach levels up to 10 Log_10_ gc/L [44], although it may vary depending on factors such as urbanization level, population served by WWTP, available infrastructures, climate conditions, and the impact of diet on the gut microbiome [44,45].

In parallel, somatic coliphages were monitored by plate count, and the results showed mean concentrations of 5.36 ± 0.79 Log_10_ plaque-forming units (pfu)/L (30/30) in influent wastewater. However, in a recent review [46], somatic coliphages were found at higher levels, with an average of 7.26 ± 0.50 Log_10_ pfu/L. Additionally, in a study [47] conducted on influent wastewater across the United States, the average of somatic coliphages was 5.61 ± 0.91 Log_10_ pfu/L.

In general, influent wastewater is known to present a high prevalence of human enteric viruses [5]. Considering the current climate change situation and the challenge of water scarcity, it is important to treat and regenerate these waters for various purposes [48]. At the international level, there are different regulations proposing acceptable removal targets for the correct reuse of wastewater [49]. Bacterial indicator counts are generally used, but monitoring of viral indicators is typically not required, though virus removal rates are often prescribed by treatment requirements for system design [50]. The most recent European legislation 2020/741 [21] sets minimum requirements for wastewater reuse, specifically requiring a ≥6 Log_10_ reduction in rotavirus and coliphages. This legislation also emphasizes the need to validate monitoring programs as a barrier to virus transmission in reclaimed water used for agricultural irrigation [21].

In reclaimed water samples (n = 30), the most prevalent virus, RV, was detected with average concentrations of 7.05 ± 0.61 Log_10_ gc/L (30/30). Additionally, HuNoV GI and HuNoV GII were found in reclaimed waters at levels of 3.23 ± 0.46 (20/30) and 6.83 ± 0.60 (17/30) Log_10_ gc/L, respectively (Figure 1). Overall, the HuNoV GI and HuNoV GII concentration in reclaimed water reported in this study was higher than those previously reported [7,16,26]. While Randazzo and collaborators [26] reported RV levels (<5.51 Log PCRU/L) lower than those reported in our study.

CrAssphage is consistently present and has been reported in waters that receive human fecal pollution [22]. All reclaimed water samples tested positive for crAssphage by qPCR, with levels ranging from 4.53 to 8.26 Log_10_ gc/L (30/30). These levels are similar to those previously described [7].

**Figure 3:**
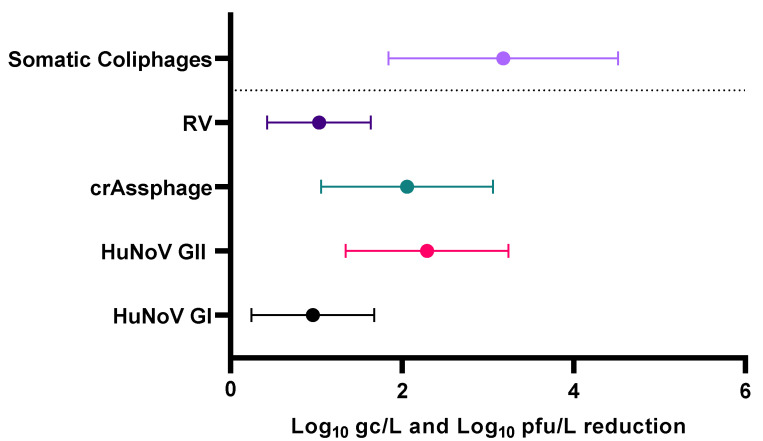
Mean reduction in human enteric viruses, crAssphage, and somatic coliphages after wastewater treatment. Abbreviations: human norovirus genotype I (HuNoV GI), human norovirus genotype II (HuNoV GII), rotavirus (RV), genome copies (gc), pfu (plaque-forming units).

The presence of somatic coliphages in reclaimed water was analyzed to verify compliance with legislative reduction requirements and to assess their correlation with the presence of human enteric viruses, as the detection of somatic coliphages in reclaimed water may serve as an indicator of the presence of enteric viruses or the efficacy of their elimination. After the wastewater treatment, the mean removal of somatic coliphages was 3.18 ± 1.74 Log_10_ pfu/L (Figure 3).

Values provided in a recent review [42] showed a reduction in somatic coliphages levels in European WWTPs of 2.32 ± 0.42 Log_10_ pfu/L, being significantly lower than the results obtained in our study. The study conducted by Worley-Morse et al. [51], carried out in United States, showed an initial mean reduction in somatic coliphages in primary treatment of 0.4 Log_10_ pfu/L. In secondary treatment, reductions ranged from 0.06 to 3 Log_10_ pfu/L, relative to initial somatic coliphages levels of 6.2 ± 0.49 Log pfu/L. While the reduction in coliphages reported in our study did not meet legislative specifications, it is noteworthy that coliphages were the only analyzed viruses to achieve complete reduction in 40% of the reclaimed water samples (Supplementary Table S2). None of the studied enteric viruses or crAssphage achieved the required reduction after the wastewater treatment (Supplementary Table S3), indicating a low efficacy in virus removal by the analyzed WWTP. The mean Log_10_ removals were 0.96 ± 0.72, 2.29 ± 0.95, 1.03 ± 0.60, and 3.18 ± 1.34 gc/L for HuNoV GI, HuNoV GII, RV, and crAssphage, respectively (Figure 3).

It is important to note that, while infectivity cannot be directly inferred from (RT)-qPCR detection, the observed combination of factors warrants caution in the reuse of these waters. Considering the levels of somatic coliphages and the high concentration of enteric viruses recorded in the reclaimed water samples of our study, it is advisable to reject these reclaimed waters and consider them unsuitable for agricultural irrigation.

### 3.2. Correlation among Enteric Viruses and Viral Indicators in Reclaimed Water

**Figure 4:**
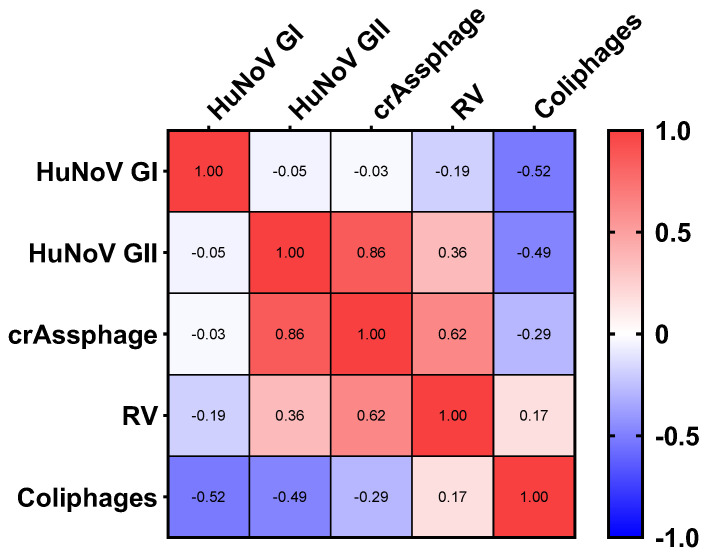
Spearman’s correlation heatmap on the presence of enteric viruses ((RT)-qPCR) and fecal contamination indicators in effluent samples. Abbreviations: human norovirus genotype I (HuNoV GI), human norovirus genotype II (HuNoV GII), rotavirus (RV).

Fecal indicator bacteria have been proven to not accurately reflect viral risk to human health [52,53] as they do for pathogenic bacteria [54]. CrAssphage, which has lately been raised as a novel fecal marker, has been suggested as a new viral indicator in wastewater samples analyses [55]. The presence of crAssphage indicates fecal contamination from human or animal sources. Increased levels of crAssphage within reclaimed water heighten the probability of pathogenic viruses. Recent studies have also shown crAssphage to be a robust indicator of fecal contamination in the environment and in different water matrices [22,23,35,36,43,44,55,56]. However, the correlation between crAssphage and the presence of human viral pathogens is not clear and further research is needed. In our study, a strong positive correlation (n = 30) of crAssphage with HuNoV GII (ρ = 0.86, *p* = 0.01) and a moderate correlation with RV (ρ = 0.62, *p* = 0.06) was observed in reclaimed water analyzed by (RT)-qPCR. The same correlation test was performed with reclaimed water samples positive for somatic coliphages and did not show any correlation in conjunction with enteric viruses (Figure 4).

### 3.3. Assessing Viral Infectivity in Influent Wastewater and Reclaimed Water by PMAxx-RT-qPCR

To avoid overestimating the risk of inactivated viruses by the use of molecular techniques, a capsid integrity assay was conducted. PCR-based monitoring of enteric viruses in reclaimed water can be a sensitive and specific tool for assessing compliance with European legislation. However, molecular-based methods can detect both infectious and non-infectious viruses, which may overestimate the risk associated with reclaimed water [26,27,28]. Traditional cell-culture methods for assessing viral infectivity in water samples have faced challenges [11], leading to the development of new methods based on capsid integrity using viability markers. These methods have shown promising results for evaluating the infectivity of enteric, mainly HuNoV and hepatitis A virus, and respiratory viruses in wastewater and other matrices [3,25,26,27,32,57,58]. Capsid integrity, among other capsid integrity methods, is a valid and robust indicator of virus infectivity and can enhance risk assessment in monitoring programs [7].

**Figure 5:**
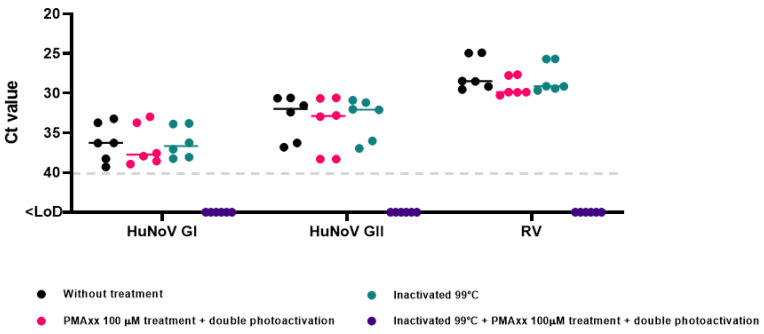
Optimization of PMAxx-RT-qPCR assay for influent wastewater samples. Abbreviations: human norovirus genotype I (HuNoV GI), human norovirus genotype II (HuNoV GII), rotavirus (RV), cycle threshold (Ct), limit of detection (LoD).

This study provides additional insights into the optimal conditions for quantifying intact capsid enteric viruses in influent wastewater and reclaimed waters, particularly for RV, for which such novel optimized methods have not been validated previously. To validate the PMAxx-RT-qPCR protocol, different dilutions of influent wastewater were conducted and were tested for HuNoV GI, HuNoV GII, and RV presence to achieve the best performance. However, the signal was not efficiently reduced after inactivation at 99 °C together with PMAxx treatment. Thus, simple photoactivation was not sufficient to evaluate the potential infectivity of HuNoV GI, HuNoV GII, and RV in these types of samples. It is known that various factors (concentration and dye intercalating conditions, matrix, among others) can prevent a proper photoactivation of PMAxx affecting signal reduction in inactivated and treated samples [26]. Therefore, diluted influent wastewater and reclaimed water samples in PBS (5-fold and 2-fold, respectively) were subjected to double photoactivation, after the thermal inactivation step, and the signal of the samples treated with PMAxx was completely reduced. In all cases, a negative process control was used (Supplementary Table S4 and Figure 5).

**Figure 6:**
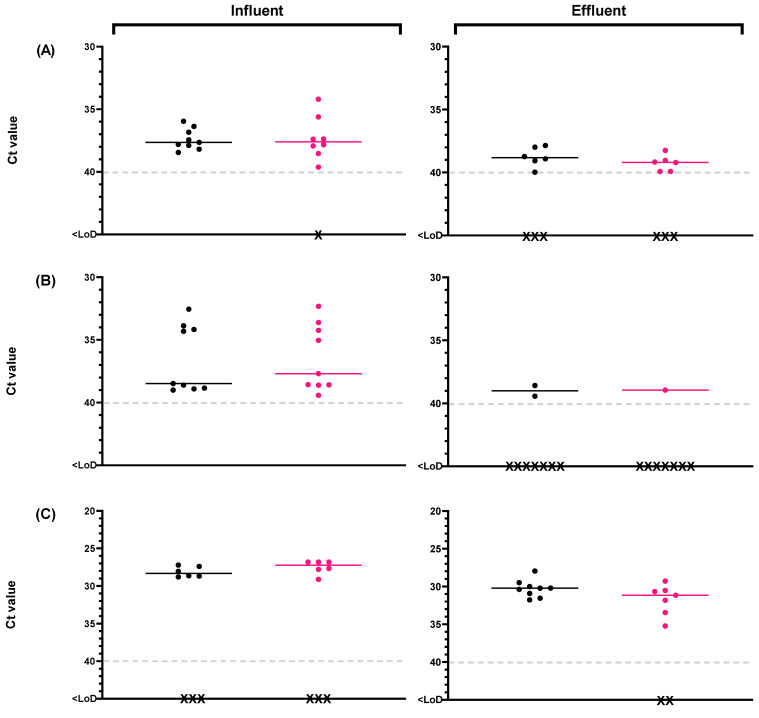
Monitoring of human enteric viruses on raw samples (black dots) and with optimized PMAxx 100 µM treatment (pink dots) in influent wastewater and reclaimed water for (**A**) human norovirus genotype I (HuNoV GI), (**B**) human norovirus genotype II (HuNoV GII), and (**C**) rotavirus (RV). Black crosses mean negative samples. Abbreviations: cycle threshold (Ct), limit of detection (LoD).

The presence of potentially infectious viruses was tested in a subset (n = 18) of influent wastewater and reclaimed water samples using the optimized PMAxx-RT-qPCR method for RV and HuNoV [32]. The evaluation of influent wastewater and reclaimed water samples over the course of the study using the PMAxx-RT-qPCR method revealed the presence of potentially infectious HuNoV GI, HuNoV GII, and RV (Supplementary Table S5). After performing the capsid integrity (RT)-qPCR with optimized conditions, the cycle threshold (Ct) is shown in Figure 6.

Our results indicate that 89% of influent wastewater treated with the optimized PMAxx protocol (n = 9) tested positive for HuNoV GI, and 100% tested positive for HuNoV GII, with an average concentration of 4.59 ± 0.32 Log_10_ gc/L (8/9) and 7.46 ± 0.50 Log_10_ gc/L (9/9). RV was present in 67% of influent wastewater samples analyzed both with and without the optimized PMAxx protocol, with higher mean levels compared to the other two viruses, at 8.12 ± 0.25 Log_10_ gc/L (6/9). In positive reclaimed water samples treated with the optimized PMAxx protocol (n = 9), HuNoV GI was detected in 67% of samples with average concentrations of 3.82 ± 0.52 Log_10_ gc/L (6/9), while HuNoV GII was only detected in one replicate of all the analyzed samples, with a concentration of 5.94 Log_10_ gc/L (1/9). Additionally, RV was detected in 78% of the samples with concentrations of 6.69 ± 0.48 Log_10_ gc/L (7/9). Results obtained after the capsid integrity assay suggest the potential spread of infectious viruses through the environment by positive reclaimed waters.

A high prevalence of HuNoV GI, GII and RV has been consistently reported in influent wastewater despite yearly fluctuations [26,59]. After reclamation treatments, enteric viruses demonstrate a significant reduction with an expected average decrease of 1 to 1.5 Log_10_ due to conventional secondary activated sludge treatment [5]. However, removal rates vary considerably based on the treatment facility [5]. In our study, the detection limit of each virus was used to perform the analyses in cases of total reduction among paired samples. The reduction in influent wastewater and reclaimed water samples mean levels using PMAxx-RT-qPCR results of HuNoV GI was 1.39 ± 0.51 Log_10_ gc/L, while HuNoV GII was detected in only one effluent sample with reduction of 3.06 ± 0.45 Log_10_ gc/L, being the enteric virus with greatest removal. Kevill et al. [60] reported values showing a similar trend to our results for HuNoV before conducting a PMAxx-RT-qPCR; however, in their case, the reductions observed for HuNoV GII were lower than those observed in our study. RV mean level reduction after the reclamation treatment was 1.29 ± 0.29 Log_10_ (Supplementary Table S6). The results of removal obtained by capsid integrity assay show statistically significant differences (*p* < 0.05) compared to those obtained from (RT)-qPCR for HuNoV GII and RV, except for HuNoV GI. This approach enables the estimation of disinfection treatment effectiveness and the risk of pathogens spreading through wastewater reuse. This fact contributes to the knowledge of HuNoV GI presenting higher resistance at reclamation and disinfection processes than HuNoV GII [39,40], having greater prevalence and stability in the environment, and therefore being more associated with water-related outbreaks and the possibility of crop contamination. Unlike HuNoV GI, HuNoV GII is generally linked to food-related outbreaks, mainly due to food handling and its lower resistance to reclamation treatments [61,62]. RV is remarkably resistant to the reclamation process, being transmitted through contaminated water among other infection pathways and being able to survive for long periods in the environment [63,64].

HuNoV GI has been reported in a high number of vegetable and fruit-associated outbreaks [65]. RV has also been detected in raw vegetables, although not as frequently as HuNoV GI [49]. Furthermore, RV has been identified as being linked to the post-harvest use of water [65,66]. However, the risk posed by RV contamination of fresh vegetables is not well understood [67]. The higher prevalence of HuNoV GI and RV in sewage indicates that reclaimed water is the probable source of fresh vegetable contamination [49,63,64,65,66]. Thus, determining the available water source quality may prevent the contamination of fresh vegetables during pre-harvest stage via irrigation and throughout the food production chain. The low infectious dose [9] of enteric viruses and their ability to remain infectious under certain conditions entails the subsequent exposure of consumers to potentially infectious HuNoV and RV by consuming fresh and uncooked vegetables. According to Regulation (EU) 2020/741 [21] and considering the detection of viruses by PMAxx-RT-qPCR, the reclaimed waters analyzed in this study should not be used for agricultural purposes.

## 4. Conclusions

In this study the monitoring of enteric viruses and crAssphage was conducted over 10 months on both influent wastewater and reclaimed water samples by (RT)-qPCR. Furthermore, an optimized capsid integrity assay was applied by using the intercalating dye PMAxx. Additionally, somatic coliphages counting was assessed and their absence in reclaimed water samples did not correlate with the removal of potential infectious viral particles.

The optimization of PMAxx-RT-qPCR method served as a useful tool to check capsid integrity and address potential infectivity of enteric viruses in both influent wastewater and reclaimed water. This study provides insights to better understand the presence and potential infectivity of enteric viruses, particularly for RV, in reclaimed waters intended for agricultural purposes. Nevertheless, capsid integrity assays do not guarantee the infectivity of the samples; therefore, future research needs to focus on comparative studies between molecular assays and viral cell culture on environmental samples.

## Data Availability

The raw data supporting the conclusions of this article will be made available by the authors on request.

## Supplementary Materials

The following supporting information can be downloaded at: https://www.mdpi.com/article/10.3390/v16060816/s1. Table S1: Primers, probes, (RT)-qPCR conditions, limit of quantification (LoQ/L) and limit of detection (LoD/L) for all targeted viruses included in this work. Table S2: Mengovirus recovery (%) and mean concentration values (Log_10_ gc/L) obtained by (RT)-qPCR of enteric viruses, crAssphage, and somatic coliphages (Log_10_ pfu/L). Table S3: Removal of enteric viruses between influent wastewater and reclaimed water (n = 9) by (RT)-qPCR. Table S4: Cycle threshold (Ct) values for the PMAxx-RT-qPCR optimization tests in influent wastewater samples. Table S5: Ct values by PMAxx-RT-qPCR for intact capsid enteric viruses and crAssphage in influent wastewater and reclaimed water. Table S6: Removal of enteric viruses in reclaimed water compared to influent wastewater (n = 9) by RT-qPCR and PMAxx-RT-qPCR.

## References

[1] Russo G.B., Goyal T., Tyler K., Thakur K.T. (2022). Re-Emergence of Poliovirus in the United States: Considerations and Implications. Ann. Neurol..

[2] Randazzo W., Truchado P., Cuevas-Ferrando E., Simón P., Allende A., Sánchez G. (2020). SARS-CoV-2 RNA in Wastewater Anticipated COVID-19 Occurrence in a Low Prevalence Area. Water Res..

[3] Canh V.D., Torii S., Yasui M., Kyuwa S., Katayama H. (2021). Capsid Integrity RT-QPCR for the Selective Detection of Intact SARS-CoV-2 in Wastewater. Sci. Total Environ..

[4] Girón-Guzmán I., Díaz-Reolid A., Truchado P., Carcereny A., García-Pedemonte D., Hernáez B., Bosch A., Pintó R.M., Guix S., Allende A. (2023). Spanish Wastewater Reveals the Current Spread of Monkeypox Virus. Water Res..

[5] Sano D., Amarasiri M., Hata A., Watanabe T., Katayama H. (2016). Risk Management of Viral Infectious Diseases in Wastewater Reclamation and Reuse: Review. Environ. Int..

[6] Farkas K., Marshall M., Cooper D., McDonald J.E., Malham S.K., Peters D.E., Maloney J.D., Jones D.L. (2018). Seasonal and Diurnal Surveillance of Treated and Untreated Wastewater for Human Enteric Viruses. Environ. Sci. Pollut. Res. Int..

[7] Cuevas-Ferrando E., Pérez-Cataluña A., Falcó I., Randazzo W., Sánchez G. (2022). Monitoring Human Viral Pathogens Reveals Potential Hazard for Treated Wastewater Discharge or Reuse. Front. Microbiol..

[8] Carter M.J. (2005). Enterically infecting viruses: Pathogenicity, transmission and significance for food and waterborne infection. J. Appl. Microbiol..

[9] Bosch A., Guix S., Sano D., Pintó R.M. (2008). New Tools for the Study and Direct Surveillance of Viral Pathogens in Water. Curr. Opin. Biotechnol..

[10] Nasser A., Sasi S., Nitzan Y. (2021). Coliphages as Indicators for the Microbial Quality of Treated Wastewater Effluents. Food Environ. Virol..

[11] Gerba C.P., Betancourt W.Q., Kitajima M., Rock C.M. (2018). Reducing Uncertainty in Estimating Virus Reduction by Advanced Water Treatment Processes. Water Res..

[12] Katayama H., Haramoto E., Oguma K., Yamashita H., Tajima A., Nakajima H., Ohgaki S. (2008). One-Year Monthly Quantitative Survey of NVes, Enteroviruses, and Adenoviruses in Wastewater Collected from Six Plants in Japan. Water Res..

[13] Prevost B., Lucas F.S., Goncalves A., Richard F., Moulin L., Wurtzer S. (2015). Large Scale Survey of Enteric Viruses in River and Waste Water Underlines the Health Status of the Local Population. Environ. Int..

[14] Santiso-Bellón C., Randazzo W., Pérez-Cataluña A., Vila-Vicent S., Gozalbo-Rovira R., Muñoz C., Buesa J., Sanchez G., Rodríguez Díaz J. (2020). Epidemiological Surveillance of Norovirus and Rotavirus in Sewage (2016–2017) in Valencia (Spain). Microorganisms.

[15] Silva-Sales M., Martínez-Puchol S., Gonzales-Gustavson E., Hundesa A., Gironès R. (2020). High Prevalence of Rotavirus A in Raw Sewage Samples from Northeast Spain. Viruses.

[16] Truchado P., Garre A., Gil M.I., Simón-Andreu P.J., Sánchez G., Allende A. (2021). Monitoring of Human Enteric Virus and Coliphages throughout Water Reuse System of Wastewater Treatment Plants to Irrigation Endpoint of Leafy Greens. Sci. Total Environ..

[17] Stobnicka-Kupiec A., Gołofit-Szymczak M., Cyprowski M., Górny R.L. (2022). Detection and Identification of Potentially Infectious Gastrointestinal and Respiratory Viruses at Workplaces of Wastewater Treatment Plants with Viability QPCR/RT-QPCR. Sci. Rep..

[18] Kumthip K., Khamrin P., Ushijima H., Maneekarn N. (2023). Detection of Six Different Human Enteric Viruses Contaminating Environmental Water in Chiang Mai, Thailand. Microbiol. Spectr..

[19] Partyka M.L., Bond R.F. (2022). Wastewater Reuse for Irrigation of Produce: A Review of Research, Regulations, and Risks. Sci. Total Environ..

[20] Anderson-Coughlin B.L., Vanore A., Shearer A.E.H., Gartley S., Joerger R.D., Sharma M., Kniel K.E. (2023). Human Norovirus Surrogates Persist in Nontraditional Sources of Irrigation Water in Excess of 100 Days. J. Food Prot..

[21] Regulation (EU) 2020/741 (2020). Regulation (EU) 2020/741 of the European Parliament and of the Council of 25 May 2020 on Minimum Requirements for Water Reuse. http://data.europa.eu/eli/reg/2020/741/oj.

[22] Farkas K., Adriaenssens E.M., Walker D.I., McDonald J.E., Malham S.K., Jones D.L. (2019). Critical Evaluation of CrAssphage as a Molecular Marker for Human-Derived Wastewater Contamination in the Aquatic Environment. Food Environ. Virol..

[23] Farkas K., Walker D.I., Adriaenssens E.M., McDonald J.E., Hillary L.S., Malham S.K., Jones D.L. (2020). Viral Indicators for Tracking Domestic Wastewater Contamination in the Aquatic Environment. Water Res..

[24] Tandukar S., Sherchan S.P., Haramoto E. (2020). Applicability of CrAssphage, Pepper Mild Mottle Virus, and Tobacco Mosaic Virus as Indicators of Reduction of Enteric Viruses during Wastewater Treatment. Sci. Rep..

[25] Leifels M., Jurzik L., Wilhelm M., Hamza I.A. (2015). Use of Ethidium Monoazide and Propidium Monoazide to Determine Viral Infectivity upon Inactivation by Heat, UV- Exposure and Chlorine. Int. J. Hyg. Environ. Health.

[26] Randazzo W., Piqueras J., Evtoski Z., Sastre G., Sancho R., Gonzalez C., Sánchez G. (2019). Interlaboratory Comparative Study to Detect Potentially Infectious Human Enteric Viruses in Influent and Effluent Waters. Food Environ. Virol..

[27] Cuevas-Ferrando E., Randazzo W., Pérez-Cataluña A., Falcó I., Navarro D., Martin-Latil S., Díaz-Reolid A., Girón-Guzmán I., Allende A., Sánchez G. (2021). Platinum Chloride-Based Viability RT-QPCR for SARS-CoV-2 Detection in Complex Samples. Sci. Rep..

[28] Canh V.D., Liu M., Sangsanont J., Katayama H. (2022). Capsid Integrity Detection of Pathogenic Viruses in Waters: Recent Progress and Potential Future Applications. Sci. Total Environ..

[29] Pérez-Cataluña A., Cuevas-Ferrando E., Randazzo W., Falcó I., Allende A., Sánchez G. (2021). Comparing Analytical Methods to Detect SARS-CoV-2 in Wastewater. Sci. Total Environ..

[30] Girón-Guzmán I., Díaz-Reolid A., Cuevas-Ferrando E., Falcó I., Cano-Jiménez P., Comas I., Pérez-Cataluña A., Sánchez G. (2023). Evaluation of Two Different Concentration Methods for Surveillance of Human Viruses in Sewage and Their Effects on SARS-CoV-2 Sequencing. Sci. Total Environ..

[31] Stachler E., Kelty C., Sivaganesan M., Li X., Bibby K., Shanks O.C. (2017). Quantitative CrAssphage PCR Assays for Human Fecal Pollution Measurement. Environ. Sci. Technol..

[32] Randazzo W., Khezri M., Ollivier J., Le Guyader F.S., Rodríguez-Díaz J., Aznar R., Sánchez G. (2018). Optimization of PMAxx Pretreatment to Distinguish between Human Norovirus with Intact and Altered Capsids in Shellfish and Sewage Samples. Int. J. Food Microbiol..

[33] (2017). Microbiology of Food and Animal Feed—Horizontal Method for Determination of Hepatitis a Virus and Norovirus in Food Using Real-time RT-PCR—Part 1. Method for Quantification.

[34] Haramoto E., Kitajima M., Hata A., Torrey J.R., Masago Y., Sano D., Katayama H. (2018). A Review on Recent Progress in the Detection Methods and Prevalence of Human Enteric Viruses in Water. Water Res..

[35] Cioffi B., Monini M., Salamone M., Pellicanò R., Di Bartolo I., Guida M., La Rosa G., Fusco G. (2020). Environmental Surveillance of Human Enteric Viruses in Wastewaters, Groundwater, Surface Water and Sediments of Campania Region. Reg. Stud. Mar. Sci..

[36] Wu H., Juel M.A.I., Eytcheson S., Aw T.G., Munir M., Molina M. (2023). Temporal and Spatial Relationships of CrAssphage and Enteric Viral and Bacterial Pathogens in Wastewater in North Carolina. Water Res..

[37] Hata A., Shirasaka Y., Ihara M., Yamashita N., Tanaka H. (2021). Spatial and Temporal Distributions of Enteric Viruses and Indicators in a Lake Receiving Municipal Wastewater Treatment Plant Discharge. Sci. Total Environ..

[38] Qiu Y., Lee B.E., Neumann N., Ashbolt N., Craik S., Maal-Bared R., Pang X.L. (2015). Assessment of Human Virus Removal during Municipal Wastewater Treatment in Edmonton, Canada. J. Appl. Microbiol..

[39] de Graaf M., Villabruna N., Koopmans M.P. (2017). Capturing Norovirus Transmission. Curr. Opin. Virol..

[40] Ibrahim C., Hammami S., Khelifi N., Pothier P., Hassen A. (2020). The Effectiveness of Activated Sludge Procedure and UV-C254 in Norovirus Inactivation in a Tunisian Industrial Wastewater Treatment Plant. Food Environ. Virol..

[41] Crank K., Li X., North D., Ferraro G.B., Iaconelli M., Mancini P., La Rosa G., Bibby K. (2020). CrAssphage Abundance and Correlation with Molecular Viral Markers in Italian Wastewater. Water Res..

[42] Ahmed W., Lobos A., Senkbeil J., Peraud J., Gallard J., Harwood V.J. (2018). Evaluation of the Novel CrAssphage Marker for Sewage Pollution Tracking in Storm Drain Outfalls in Tampa, Florida. Water Res..

[43] Malla B., Makise K., Nakaya K., Mochizuki T., Yamada T., Haramoto E. (2019). Evaluation of Human- and Animal-Specific Viral Markers and Application of CrAssphage, Pepper Mild Mottle Virus, and Tobacco Mosaic Virus as Potential Fecal Pollution Markers to River Water in Japan. Food Environ. Virol..

[44] Sabar M.A., Honda R., Haramoto E. (2022). CrAssphage as an Indicator of Human-Fecal Contamination in Water Environment and Virus Reduction in Wastewater Treatment. Water Res..

[45] Honap T.P., Sankaranarayanan K., Schnorr S.L., Ozga A.T., Warinner C., Jr C.M.L. (2020). Biogeographic Study of Human Gut-Associated CrAssphage Suggests Impacts from Industrialization and Recent Expansion. PLoS ONE.

[46] Kelmer A.R., Ramos R., Dias H.O. (2023). Coliphages as Viral Indicators in Municipal Wastewater: A Comparison Between the ISO and the USEPA Methods Based on a Systematic Literature Review. Water Res..

[47] Korajkic A., McMinn B., Herrmann M.P., Sivaganesan M., Kelty C.A., Clinton P., Nash M.S., Shanks O.C. (2020). Viral and Bacterial Fecal Indicators in Untreated Wastewater across the Contiguous United States Exhibit Geospatial Trends. Appl. Environ. Microbiol..

[48] UN Water (2018). Sustainable Development Goal 6: Synthesis Report 2018 on Water and Sanitation.

[49] Fernandes L.S., Galvão A., Santos R., Monteiro S. (2023). Impact of Water Reuse on Agricultural Practices and Human Health. Environ. Res..

[50] (2012). Guidelines for Water Reuse.

[51] Worley-Morse T., Mann M., Khunjar W., Olabode L., Gonzalez R. (2019). Evaluating the fate of bacterial indicators, viral indicators, and viruses in water resource recovery facilities. Water Environ. Res..

[52] Harwood V.J., Staley C., Badgley B.D., Borges K., Korajkic A. (2014). Microbial Source Tracking Markers for Detection of Fecal Contamination in Environmental Waters: Relationships between Pathogens and Human Health Outcomes. FEMS Microbiol. Rev..

[53] Ballesté E., Pascual-Benito M., Martín-Díaz J., Blanch A.R., Lucena F., Muniesa M., Jofre J., García-Aljaro C. (2019). Dynamics of CrAssphage as a Human Source Tracking Marker in Potentially Faecally Polluted Environments. Water Res..

[54] Truchado P., Gil M.I., López C., Garre A., López-Aragón R.F., Böhme K., Allende A. (2021). New Standards at European Union Level on Water Reuse for Agricultural Irrigation: Are the Spanish Wastewater Treatment Plants Ready to Produce and Distribute Reclaimed Water within the Minimum Quality Requirements?. Int. J. Food Microbiol..

[55] Hamza I.A., Abd-Elmaksoud S. (2023). Applicability of CrAssphage as a Performance Indicator for Viral Reduction during Activated Sludge Wastewater Treatment. Environ. Sci. Pollut. Res..

[56] Threndyle R.E., Kurylyk B.L., Huang Y., Johnston L.H., Jamieson R.C. (2022). CrAssphage as an Indicator of Groundwater-Borne Pollution in Coastal Ecosystems. Environ. Res. Commun..

[57] Shirasaki N., Matsushita T., Matsui Y., Koriki S. (2020). Suitability of Pepper Mild Mottle Virus as a Human Enteric Virus Surrogate for Assessing the Efficacy of Thermal or Free-Chlorine Disinfection Processes by Using Infectivity Assays and Enhanced Viability PCR. Water Res..

[58] Canh V.D., Torii S., Furumai H., Katayama H. (2021). Application of Capsid Integrity (RT-)QPCR to Assessing Occurrence of Intact Viruses in Surface Water and Tap Water in Japan. Water Res..

[59] Eftim S.E., Hong T., Soller J., Boehm A., Warren I., Ichida A., Nappier S.P. (2017). Occurrence of Norovirus in Raw Sewage—A Systematic Literature Review and Meta-Analysis. Water Res..

[60] Kevill J.L., Farkas K., Ridding N., Woodhall N., Malham S.K., Jones D.L. (2024). Use of Capsid Integrity-qPCR for Detecting Viral Capsid Integrity in Wastewater. Viruses.

[61] Nordgren J., Matussek A., Mattsson A., Svensson L., Lindgren P.-E. (2009). Prevalence of Norovirus and Factors Influencing Virus Concentrations during One Year in a Full-Scale Wastewater Treatment Plant. Water Res..

[62] da Silva A.K., Le Saux J.-C., Parnaudeau S., Pommepuy M., Elimelech M., Le Guyader F.S. (2007). Evaluation of Removal of Noroviruses during Wastewater Treatment, Using Real-Time Reverse Transcription-PCR: Different Behaviors of Genogroups I and II. Appl. Environ. Microbiol..

[63] Kotwal G., Cannon J.L. (2014). Environmental Persistence and Transfer of Enteric Viruses. Curr. Opin. Virol..

[64] Omatola C.A., Olaniran A.O. (2022). Epidemiological Significance of the Occurrence and Persistence of Rotaviruses in Water and Sewage: A Critical Review and Proposal for Routine Microbiological Monitoring. Environ. Sci. Process Impacts.

[65] Koutsoumanis K., Ordóñez A.A., Bolton D., Bover-Cid S., Chemaly M., De Cesare A., Herman L., Hilbert F., Lindqvist R., EFSA Panel on Biological Hazards (BIOHAZ) (2023). Microbiological hazards associated with the use of water in the post-harvest handling and processing operations of fresh and frozen fruits, vegetables and herbs (ffFVHs). Part 1 (outbreak data analysis, literature review and stakeholder questionnaire). EFSA J..

[66] Quiroz-Santiago C., Vázquez-Salinas C., Natividad-Bonifacio I., Barrón-Romer B.L., Quiñones-Ramírez E.I. (2014). Rotavirus G2P [4] Detection in Fresh Vegetables and Oysters in Mexico City. J. Food Prot..

[67] Fuzawa M., Smith R.L., Ku K.M., Shisler J.L., Feng H., Juvik J.A., Nguyen T.H. (2020). Roles of Vegetable Surface Properties and Sanitizer Type on Annual Disease Burden of Rotavirus Illness by Consumption of Rotavirus-Contaminated Fresh Vegetables: A Quantitative Microbial Risk Assessment. Risk Anal..

