# Supplementary material for: Capsid Integrity Detection of Enteric Viruses in Reclaimed Waters
Source: Viruses. 2024 May 21;16(6):816. doi: 10.3390/v16060816 (PMC11209584; doi:10.3390/v16060816)
Supplement: Supplementary file 1 [file viruses-16-00816-s001.zip › viruses-3011985-supplementary.pdf]

SUPPLEMENTARY MATERIAL

Capsid integrity detection of enteric viruses in reclaimed waters

Pablo Puchades-Colera, Azahara Díaz-Reolid, Inés Girón-Guzmán, Enric Cuevas-Ferrando, Alba Pérez-Cataluña, Gloria Sánchez*.

VISAFELab. Department of Preservation and Food Safety Technologies. Institute of Agrochemistry and Food Technology. IATA-CSIC. Av. Agustín Escardino 7. Paterna. 46980. Valencia. Spain.

*Corresponding author: gloriasanchez@iata.csic.es

Table S1. Primers, probes, (RT)-qPCR conditions, limit of quantification (LoQ/L) and limit of detection (LoD/L) for all targeted viruses included in this work. Abbreviations: human norovirus genotype I (HuNoV GI), human norovirus genotype II (HuNoV GII), rotavirus (RV), MgV (mengovirus), limit of quantification (LoQ), limit of detection (LoD).

Virus	Primers and Probe	Sequence	(RT)-qPCR conditions	LoQ/L	LoD/L	Reference
**HuNoV GI**	QNIF4	CGC TGG ATG CGN TTC CAT	RT: 55 °C for 60 min. Preheating: 95 °C for 5 min PCR (45 cycles) 95 °C for 15 s. 60 °C for 60 s. 65 °C for 60 s	8.77x10^3	2.77x10^3	
	NV1LCR	CCT TAG ACG CCA TCA TCA TTT AC				
	NVGG1p	[FAM]-TGG ACA GGA GAY CGC RAT CT-[BHQ]				
**HuNoV GII**	QNIF2	ATG TTC AGR TGG ATG AGR TTC TCW GA	RT: 55 °C for 60 min. Preheating: 95 °C for 5 min PCR (45 cycles) 95 °C for 15 s. 60 °C for 60 s. 65 °C for 60 s.	2.12x10^4	1.60x10^4	(33)
	COG2R	TCG ACG CCA TCT TCA TTC ACA				
	QNIFS	[FAM]-AGC ACG TGG GAG GGC GAT CG-[BHQ]				
**MgV**	Mengo110	GCG GGT CCT GCC GAA AGT	RT: 55 °C for 60 min. Preheating: 95 °C for 5 min PCR (45 cycles) 95 °C for 15 s. 60 °C for 60 s. 65 °C for 60 s.	2.07x10^4	1.46x10^4	
	Mengo209	GAA GTA ACA TAT AGA CAG ACG CAC AC				
	Mengo147	[FAM]-ATC ACA TTA CTG GCC GAA GC-[MGBNFQ]				
**RV**	JVKF	CAG TGG TTG ATG CTC AAG ATG GA	RT: 50 °C for 30 min. Preheating: 95 °C for 15 min PCR (45 cycles) 94 °C for 10 s. 55 °C for 30 s. 72 °C for 20 s.	1.71x10^4	4.06x10^3	(70)
	JVKR	TCA TTG TAA TCA TAT TGA ATA CCC A				
	JVKP	[FAM]-ACA ACT GCA GCT TCA AAA GAA GWG T-[BHQ]				
**crAssphage**	064F1	TGT ATA GAT GCT GCT GCA ACT GTA CTC	Preheating: 95 °C for 5 min PCR (45 cycles) 95 °C for 5 s. 60 °C for 30 s	1.64x10^5	4.93x10^4	(31)
	064R	CGT TGT TTT CAT CTT TAT CTT GTC CAT				
	064P1	[FAM]-CTG AAA TTG TTC ATA AGC AA-[MGBNFQ]				

Table S2. Mengovirus recovery (%) and mean concentration values (Log_{10} gc/L) obtained by (RT)-qPCR of enteric viruses, crAssphage, and somatic coliphages (Log_{10} pfu/L). Abbreviations: human norovirus genotype I (HuNoV GI), human norovirus genotype II (HuNoV GII), rotavirus (RV), MgV (mengovirus), genome copies (gc), pfu (plaque forming units), limit of detection (LoD).

Month	Sample type	HuNoV GI (Log_{10} gc/L)	HuNoV GII (Log_{10} gc/L)	crAssphage (Log_{10} gc/L)	RV (Log_{10} gc/L)	Somatic coliphages (Log_{10} pfu/L)	MgV Recovery (%)
**May**	Influent	5.10 ± 0.28	8.03 ± 0.08	7.94 ± 0.34	8.77 ± 0.02	6.76 ± 0.02	12.71
	Effluent	2.94 ± 0.00	6.29 ± 0.05	6.11 ± 0.02	7.78 ± 0.17	1.90 ± 0.08	36.63
**June**	Influent	3.91 ± 0.23	7.06 ± 0.01	8.69 ± 0.03	8.10 ± 0.03	6.29 ± 0.05	38.23
	Effluent	2.86 ± 0.00	<LoD	4.53 ± 0.18	6.74 ± 0.01	0.70 ± 0.00	29.00
	Influent	5.27 ± 0.04	8.36 ± 0.06	8.38 ± 0.11	8.10 ± 0.03	6.24 ± 0.01	19.14
	Effluent	<LoD	<LoD	6.88 ± 0.53	6.74 ± 0.01	1.88 ± 0.49	25.49
**July**	Influent	4.33 ± 0.04	8.25 ± 0.04	7.59 ± 0.10	7.72 ± 0.00	6.39 ± 0.11	29.14
	Effluent	2.75 ± 0.00	6.24 ± 0.02	5.60 ± 0.31	5.96 ± 0.04	1.88 ± 0.31	26.14
	Influent	5.06 ± 0.08	8.32 ± 0.02	8.81 ± 0.11	8.71 ± 0.01	5.64 ± 0.01	10.83
	Effluent	3.96 ± 0.11	5.92 ± 0.01	5.70 ± 0.09	7.06 ± 0.06	1.70 ± 0.12	18.88
**September**	Influent	4.52 ± 0.08	7.78 ± 0.01	9.67 ± 0.18	8.03 ± 0.02	5.22 ± 0.02	20.37
	Effluent	3.61 ± 0.00	<LoD	6.77 ± 0.02	6.61 ± 0.00	1.81 ± 0.25	61.31
	Influent	<LoD	5.65 ± 0.01	9.21 ± 0.26	7.11 ± 0.02	5.61 ± 0.01	15.47
	Effluent	<LoD	6.56 ± 0.08	6.08 ± 0.11	6.36 ± 0.15	1.00 ± 0.00	54.48
**October**	Influent	<LoD	8.35 ± 0.00	8.19 ± 0.00	8.20 ± 0.01	5.72 ± 0.01	28.97
	Effluent	<LoD	6.17 ± 0.09	5.76 ± 0.07	6.42 ± 0.08	1.30 ± 0.34	29.80
	Influent	4.26 ± 0.11	9.14 ± 0.02	8.19 ± 0.06	8.40 ± 0.00	5.72 ± 0.12	22.70
	Effluent	2.81 ± 0.00	6.52 ± 0.05	5.76 ± 0.17	7.04 ± 0.11	1.30 ± 0.34	19.34
	Influent	<LoD	9.23 ± 0.13	8.26 ± 0.13	8.33 ± 0.03	5.61 ± 0.03	63.64
	Effluent	<LoD	6.98 ± 0.04	5.49 ± 0.71	7.21 ± 0.03	3.51 ± 0.01	53.40
	Influent	4.25 ± 0.07	8.86 ± 0.00	8.45 ± 0.03	8.01 ± 0.17	5.84 ± 0.05	45.35
	Effluent	<LoD	7.41 ± 0.00	5.94 ± 0.29	6.30 ± 0.01	1.88 ± 0.04	70.93
**November**	Influent	3.92 ± 0.00	8.82 ± 0.02	9.06 ± 0.22	7.97 ± 0.04	5.18 ± 0.00	54.04
	Effluent	3.83 ± 0.02	6.66 ± 0.10	5.84 ± 0.16	6.50 ± 0.13	1.18 ± 0.21	62.81
	Influent	3.74 ± 0.20	8.43 ± 0.05	8.82 ± 0.15	7.00 ± 0.03	5.27 ± 0.02	38.63
	Effluent	3.56 ± 0.03	6.75 ± 0.01	6.00 ± 0.21	6.49 ± 0.00	1.30 ± 0.00	22.10
	Influent	4.25 ± 0.00	8.57 ± 0.05	8.51 ± 0.12	8.10 ± 0.00	4.93 ± 0.19	22.30
	Effluent	3.68 ± 0.04	6.58 ± 0.00	6.45 ± 0.27	6.93 ± 0.07	1.30 ± 0.00	22.61
	Influent	3.74 ± 0.02	8.50 ± 0.07	8.89 ± 0.11	8.11 ± 0.04	4.98 ± 0.03	30.91
	Effluent	3.97 ± 0.00	<LoD	5.89 ± 0.03	6.21 ± 0.13	<LoD	28.97
	Influent	3.90 ± 0.00	9.08 ± 0.16	7.35 ± 0.07	8.32 ± 0.00	5.66 ± 0.10	49.43
	Effluent	3.69 ± 0.00	8.13 ± 0.01	8.26 ± 0.12	8.15 ± 0.02	<LoD	35.62
**December**	Influent	3.54 ± 0.00	8.37 ± 0.09	9.05 ± 0.27	8.11 ± 0.03	3.81 ± 0.05	37.41
	Effluent	3.57 ± 0.16	8.04 ± 0.11	8.15 ± 0.11	8.20 ± 0.03	<LoD	37.36
	Influent	2.79 ± 0.00	5.81 ± 0.00	5.76 ± 0.03	7.17 ± 0.03	5.76 ± 0.00	47.20
	Effluent	2.92 ± 0.00	<LoD	5.89 ± 0.02	7.24 ± 0.13	<LoD	53.47
**January**	Influent	3.24 ± 0.00	8.31 ± 0.03	8.33 ± 0.00	8.35 ± 0.00	5.18 ± 0.15	28.58
	Effluent	2.77 ± 0.00	7.01 ± 0.01	6.19 ± 0.07	7.14 ± 0.00	1.40 ± 0.12	68.64
	Influent	<LoD	8.52 ± 0.02	9.19 ± 0.25	8.72 ± 0.03	5.76 ± 0.00	39.65
	Effluent	<LoD	6.90 ± 0.26	6.66 ± 0.16	6.79 ± 0.00	<LoD	36.05
	Influent	3.74 ± 0.00	8.51 ± 0.10	8.85 ± 0.22	8.14 ± 0.01	5.65 ± 0.06	21.12
	Effluent	<LoD	6.88 ± 0.12	6.65 ± 0.02	7.19 ± 0.06	<LoD	15.47
	Influent	4.32 ± 0.05	7.65 ± 0.01	8.93 ± 0.04	<LoD	3.81 ± 0.06	21.68
	Effluent	3.06 ± 0.00	<LoD	7.03 ± 0.00	6.72 ± 0.00	<LoD	43.43
**February**	Influent	4.89 ± 0.00	7.65 ± 0.03	8.93 ± 0.00	<LoD	5.65 ± 0.21	8.08
	Effluent	2.73 ± 0.00	<LoD	6.72 ± 0.05	6.72 ± 0.00	<LoD	21.50
	Influent	4.38 ± 0.00	7.58 ± 0.03	8.41 ± 0.04	<LoD	4.45 ± 0.10	30.44
	Effluent	<LoD	<LoD	6.54 ± 0.05	6.85 ± 0.16	<LoD	77.46
	Influent	4.98 ± 0.07	7.93 ± 0.03	8.91 ± 0.04	8.14 ± 0.03	4.43 ± 0.07	22.59
	Effluent	2.47 ± 0.00	7.02 ± 0.00	7.24 ± 0.03	7.04 ± 0.12	<LoD	79.94
**March**	Influent	3.71 ± 0.36	6.63 ± 0.00	8.37 ± 0.06	8.17 ± 0.01	5.72 ± 0.06	9.07
	Effluent	<LoD	<LoD	6.61 ± 0.09	7.82 ± 0.07	<LoD	45.03
	Influent	4.21 ± 0.05	6.66 ± 0.00	8.16 ± 0.04	8.53 ± 0.04	5.19 ± 0.06	11.95

Effluent	3.10 ± 0.00	<LoD	6.86 ± 0.13	7.87 ± 0.02	1.65 ± 0.21	28.64
Influent	3.99 ± 0.02	6.75 ± 0.08	8.47 ± 0.09	8.37 ± 0.04	5.65 ± 0.09	9.23
Effluent	<LoD	<LoD	6.54 ± 0.06	7.77 ± 0.03	1.58 ± 0.39	11.72
Influent	3.12 ± 0.21	6.73 ± 0.02	8.09 ± 0.05	8.21 ± 0.02	3.48 ± 0.49	13.24
Effluent	3.10 ± 0.00	<LoD	6.93 ± 0.08	8.00 ± 0.08	<LoD	99.20
Influent	3.80 ± 0.00	6.65 ± 0.01	8.01 ± 0.00	8.21 ± 0.00	3.45 ± 0.07	18.49
Effluent	3.23 ± 0.00	<LoD	6.68 ± 0.06	7.66 ± 0.01	<LoD	27.14

Table S3. Removal of enteric viruses between influent wastewater and reclaimed water (n=9) by (RT)-qPCR. The reductions that were calculated using the LoD were marked with an (*). The cells left empty indicate that no removal values were calculated due to higher levels of reclaimed water compared to influent wastewater. Reclaimed water that was positive out of the total tested is shown in parentheses. Abbreviations: human norovirus genotype I (HuNoV GI), human norovirus genotype II (HuNoV GII), rotavirus (RV), genome copies (gc), pfu (plaque forming units), limit of detection (LoD).

**Month**	**HuNoV GI (Log_{10} gc/L)** (LoD/L= $2.77x10^3$) **(17/30)**	**HuNoV GII (Log_{10} gc/L)** (LoD/L= $1.60x10^4$) **(16/30)**	**RV (Log_{10} gc/L)** (LoD/L= $4.06x10^3$) **(25/30)**	**crAssphage (Log_{10} gc/L)** (LoD/L= $4.93x10^4$) **(28/30)**	**Somatic coliphages (Log_{10} pfu/L)** (LoD/L= $1x10^3$) **(18/30)**
**May**	2.16	1.74	0.99	1.82	4.86
**June**	1.05	2.86*	1.35	4.16	5.59
	1.83*	4.16*	1.35	1.50	4.36
**July**	1.57	2.01	1.76	1.98	4.51
	1.09	2.40	1.65	3.11	3.94
**September**	0.92	3.58*	1.42	2.91	3.40
	<LoD	-	0.76	3.14	4.61
**October**	<LoD	2.18	1.77	2.44	4.41
	1.45	2.63	1.36	2.44	4.41
	<LoD	2.25	1.12	2.77	2.10
	0.81*	1.45	1.71	2.51	3.96
**November**	0.08	2.16	1.46	3.22	4.01
	0.18	1.68	0.51	2.82	3.97
	0.57	1.98	1.17	2.06	3.63
	-	4.30*	1.90	3.00	1.98*
	0.21	0.95	0.17	-	2.66*
**December**	-	0.33	-	0.90	0.81*
	-	1.61*	-	-	2.76*
**January**	0.46	1.30	1.22	2.14	3.79
	<LoD	1.62	1.94	2.53	2.76*
	0.30*	1.63	0.95	2.20	3.54
	1.25	3.45*	-	1.90	2.65*
**February**	2.16	3.45*	-	2.21	2.65*
	0.94*	3.38*	-	1.86	1.45*
	2.51	0.91	1.10	1.66	1.33*
**March**	0.27*	2.43*	0.36	1.76	2.72*
	1.11	2.46*	0.66	1.30	3.54

0.55*	2.55*	0.59	1.93	4.06
0.02	2.53*	0.20	1.16	0.48*
0.57	2.45*	0.55	1.32	0.45*

Table S4. Cycle threshold (Ct) values for the PMAxx-RT-qPCR optimization tests in influent wastewater samples. Abbreviations: human norovirus genotype I (HuNoV GI), human norovirus genotype II (HuNoV GII), rotavirus (RV), cycle threshold (Ct), limit of detection (LoD).

	HuNoV GI (Ct)			
**Influent concentrate (5-fold diluted)**	**Ø**	**PMAxx 100µM**	**99°C**	**99°C + PMAxx 100µM**
	36.17 ± 2.40	36.61 ± 2.59	36.21 ± 1.97	<LoD
	**HuNoV GII (Ct)**			
**Influent concentrate (5-fold diluted)**	**Ø**	**PMAxx 100µM**	**99°C**	**99°C + PMAxx 100µM**
	33.04 ± 2.79	33.94 ± 3.53	33.19 ± 2.61	<LoD
	**RV (Ct)**			
**Influent concentrate (5-fold diluted)**	**Ø**	**PMAxx 100µM**	**99°C**	**99°C + PMAxx 100µM**
	27.59 ± 2.10	29.22 ± 1.18	28.11 ± 1.90	<LoD

Table S5. Ct values by PMAxx-RT-qPCR for intact capsid enteric viruses and crAssphage in influent wastewater and reclaimed water. Abbreviations: human norovirus genotype I (HuNoV GI), human norovirus genotype II (HuNoV GII), rotavirus (RV), cycle threshold (Ct), limit of detection (LoD).

**Month**	**Type sample**	**HuNoV GI (Ct)**	**HuNoV GII(Ct)**	**crAssphage (Ct)**	**RV (Ct)**
**January**	Influent	38.54 ± 1.32	35.04 ± 0.04	26.95 ± 0.08	<LoD
	Effluent	<LoD	<LoD	33.09 ± 0.58	<LoD
**February**	Influent	<LoD	34.24 ± 0.09	27.91 ± 0.09	<LoD
	Effluent	<LoD	<LoD	34.29 ± 0.21	<LoD
	Influent	35.61 ± 0.15	33.62 ± 0.04	27.24 ± 0.19	<LoD
	Effluent	<LoD	<LoD	34.31 ± 0.14	35.22
	Influent	34.20 ± 0.23	32.32 ± 0.30	25.25 ± 0.05	27.77 ± 0.06
	Effluent	39.04	38.94	32.04 ± 0.08	33.45 ± 0.39
**March**	Influent	37.83 ± 0.49	39.41	26.95 ± 0.17	29.10 ± 0.68
	Effluent	39.91	<LoD	33.36 ± 0.37	31.82 ± 0.00
	Influent	37.39 ± 0.92	38.61 ± 0.69	25.22 ± 0.18	26.80 ± 0.00
	Effluent	39.21 ± 0.89	<LoD	31.53 ± 0.15	30.52 ± 0.08
	Influent	37.93 ± 0.52	37.68 ± 0.28	25.59 ± 0.11	26.80 ± 0.10
	Effluent	39.92	<LoD	32.36 ± 0.03	30.66 ± 0.00
	Influent	39.62	38.56 ± 0.58	26.98 ± 0.05	26.80 ± 0.00
	Effluent	38.25	<LoD	30.81 ± 0.18	29.28 ± 0.18
	Influent	37.37 ± 0.26	38.58 ± 0.12	25.18 ± 0.06	27.65 ± 0.00
	Effluent	39.17	<LoD	32.52 ± 0.93	31.16 ± 0.00

Table S6. Removal of enteric viruses in reclaimed water compared to influent wastewater (n=9) by RT-qPCR and PMAxx-RT-qPCR. Reductions calculated using the limit of detection (LoD) are marked with an (*). Reclaimed water testing positive out of the total tested is shown in parentheses. Abbreviations: human norovirus genotype I (HuNoV GI), human norovirus genotype II (HuNoV GII), rotavirus (RV).

**HuNoV GI (Log$_{10}$ gc/L)** (LoD/L= 2.77x10^3)		**HuNoV GII (Log$_{10}$ gc/L)** (LoD/L= 1.60x10^4)		**RV (Log$_{10}$ gc/L)** (LoD/L= 4.06x10^3)	
**RT-qPCR (6/9)**	**PMAxx-RT-qPCR (6/9)**	**RT-qPCR (1/9)**	**PMAxx-RT-qPCR (1/9)**	**RT-qPCR (6/9)**	**PMAxx-RT-qPCR (7/9)**
1.25	1.20*	0.79*	3.11*	<LoD	<LoD
2.16	0.33*	3.45*	3.45*	<LoD	<LoD
0.94*	0.87*	3.38*	3.49*	<LoD	<LoD
2.51	0.90	0.91	2.39	1.10	1.74
0.27*	0.99	2.43*	3.14*	0.36	1.09
1.11	0.91	2.46*	3.28*	0.66	1.34
0.55*	1.97	2.55*	3.36*	0.59	1.47
0.02	0.02	2.53*	2.22*	0.20	1.07
0.57	0.10	2.45*	3.12*	0.55	1.01
